# Essential tensions in infant rearing

**DOI:** 10.1093/emph/eou007

**Published:** 2014-04-08

**Authors:** Katie Hinde

**Affiliations:** Department of Human Evolutionary Biology, Harvard University, Cambridge, MA; Nutrition Laboratory, Smithsonian National Zoological Park, Washington, DC; Brain, Mind, and Behavior Unit, California National Primate Research Center, Davis CA, USA

**Keywords:** lactation, co-sleeping, infant development, maternal care

The mother–infant dynamic is an elaborate negotiation due to the essential tension generated by their divergent interests. Natural selection has favored adaptations in primate mothers to allocate resources somewhat equally among a series of offspring at the same time favoring adaptations in individual young to operate with greater self-interest. This parent–offspring conflict, first conceptualized by Trivers in 1974 [1], continues to motivate extensive research. How wonderful to see David Haig renewing consideration of infant sleep patterning as an extrapolation of parent–offspring conflict first posed by Blurton-Jones and da Costa 25 years ago [2, 3]. Human and non-human primate infants have a wide repertoire of behavioral tactics to motivate care-giving from their mother including demands, risk-taking and tantrums. In this way, infants are agents in their own development and are able to, in part, shape maternal effort [4] and potentially influence mother’s subsequent reproduction. Given this context of simultaneous coordination and conflict between mother and infant, distinguishing honest signals of infant need from self-interested, care-extracting signals poses a challenge. Similarly difficult is disentangling whether ‘troubled infant sleep’ is an adaptation to extend the mother’s lactational amenorrhea or is a reflection of other aspects of infant metabolic priorities, developmental transitions and a modern milieu.

Among ‘WEIRD’ populations (western, educated, industrialized, rich and democratic [5]), fragmented sleep seemingly emerges around 6 months of age [2]. At 6 months, the metabolic needs of the human infant will soon eclipse the capacity of the mammary gland to synthesize milk and more frequent feedings may be necessary to sustain developmental trajectories. Breast milk is relatively dilute, a hallmark of a primate heritage of slow growth rates and frequent suckling bouts [6]. Does infant demand for night feedings function to satisfy metabolic requirements or to increase maternal metabolic load to extend IBI, or both simultaneously, or in turn across time? These remain empirically open questions. For individuals adhering to current WHO recommendations, 6 months also mark the introduction of complementary foods [7] precipitating dramatic restructuring of the microbial ecology of the infant’s gastrointestinal tract [8, 9]. The emergent field of microbial endocrinology is now tackling the bi-directional signaling between the brain and intestinal bacteria through the gut–brain axis [10, 11]. Alterations of the microbial community ecology can cause intestinal discomfort, and in rodent models, can increase anxiety-like behavior [10, 11]. Both may increase night waking and comfort-seeking behaviors in the infant.

Importantly, just as infants are active agents in the maternal care they elicit, they can also affect the milk their mothers synthesize. Even though milk is a ‘maternal product’ [2], this does not mean that milk synthesis is always at the mother’s evolutionary optimum. Maternal milk synthesis, as with other aspects of parenting, is vulnerable to the negotiations of parent–offspring conflict. For example, infant suckling intensity and demand can influence milk synthesis [12]. Most recently, it has emerged that fetal signals influence milk synthesis dynamically across lactations in cows in ways that have an impact on maternal investment in current and future offspring, and similar effects are likely operating in humans [13]. As suggested by Haig, paternally imprinted genes [2] in the offspring may influence the milk a mother synthesizes through fetal signaling as well as through behavioral demand post-natally. There is also mounting evidence that some milk constituents are likely to influence infant metabolism and behavioral phenotype in the mother’s interest [14].

Most of us now live far removed from the ancestral conditions in which humans evolved; the small-scale societies of hunter–gatherers foraging across a mosaic landscape [15]. However, cross-culturally, many infants are developing in the adaptively relevant environment (ARE) of contingent interactions with their mother. Close contact with the mother (and others) provides nourishment, thermoregulation and socioemotional support. At night, when foraging activities and social interactions are suspended, primate infants generally have uninterrupted access to mother’s milk [16]. Among Wied’s marmosets (*Callithrix kuhlii*), sleep is disturbed three times more in females with infants than in females without infants, particularly in the first 2 weeks post-partum [17]. Wied’s marmosets gestate for ∼143 days and have an inter-birth interval of ∼150 days [18]. Taken together, these studies indicate that their most disturbed sleep is concurrent with post-partum ovulation and conception of the subsequent litter. Milk synthesis, infant care, disturbed sleep and subsequent conception are compatible in these small-bodied marmosets. They have a number of interesting adaptations for high reproductive output, so extrapolating to humans is constrained, but these data do inspire some caution in attributing night nursing as a tactic to specifically inhibit ovulation in mothers. In a majority of human cultures for which data are available, mothers and infants sleep in close proximity, and often on the same sleep surface [16]. Night-time breastfeeding interactions between mothers and infants, facilitated by safe co-sleeping, may reduce the risk of SIDS [16]. The extent to which infants survive and thrive will be a potent target of selection. Night nursing may function to improve infant outcomes not necessarily through extending the mother’s inter-birth interval, but by directly providing nourishment and protection consistently in a 24-h period.

Increasingly, aspects of modern parenting diverge from the ARE of infancy, notably the use of breast-milk alternatives, artificial expression of breast milk [19] and sleeping apart [16]. These will alter both the coordination and conflict between mother and infant and may increase the magnitude of sleep disruption in mothers. For example, in the absence of a safe co-sleeping arrangement [16], infant signals to suckle at night are often, and perhaps necessarily, amplified because the more subtle signaling between mother and infant are unavailable. Moreover, the consolidated sleep of WEIRD adults diverges from the segmented sleep described in other cultures [20]. Worthman reports that in a cross-cultural sample of foragers, horticulturalists, pastoralists and agriculturists that ‘sleep settings offered rich and dynamic sensory properties’. As a result sleep is often social, flexible and interrupted throughout the life span [20]. The expectation that mothers and infants ‘should’ have uninterrupted, consolidated sleep is, in many ways, a historical artifact [16]. When we embrace an evolutionary perspective to understand human health and behavior, we can gain crucial insights, especially when we focus that lens on our baseline expectations.

**Conflict of interest:** None declared.
